# Type 2 and type 3 innate lymphoid cells at the maternal-fetal interface: implications in preterm birth

**DOI:** 10.1186/s12865-021-00423-x

**Published:** 2021-05-06

**Authors:** João Mendes, Paulo Rodrigues-Santos, Ana Luísa Areia, Jani-Sofia Almeida, Vera Alves, Manuel Santos-Rosa, Anabela Mota-Pinto

**Affiliations:** 1 Faculty of Medicine, Coimbra Institute for Clinical and Biomedical Research (iCBR), University of Coimbra, Polo III - Health Sciences Campus, Azinhaga Santa Comba, Celas, 3000-548 Coimbra, Portugal; 2 Faculty of Medicine, Center of Investigation in Environment, Genetics and Oncobiology (CIMAGO), University of Coimbra, Apartado 9015, 3001-301 Coimbra, Portugal; 3 General Pathology Institute, Faculty of Medicine, University of Coimbra, Rua Larga, 3004-504 Coimbra, Portugal; 4 Faculty of Medicine (FMUC), Institute of Immunology, University of Coimbra, Rua Larga, 3004-504 Coimbra, Portugal; 5 Center for Neuroscience and Cell Biology (CNC), Laboratory of Immunology and Oncology, University of Coimbra, Rua Larga, 3004-504 Coimbra, Portugal; 6 Center for Innovation in Biomedicine and Biotechnology (CIBB), University of Coimbra, Polo III - Health Sciences Campus Azinhaga Santa Comba, Celas, 3000-548 Coimbra, Portugal; 7 Coimbra Hospital and Universitary Centre (CHUC), Obstetrics Unit, R. Miguel Torga 1, 3030-165 Coimbra, Portugal

**Keywords:** Preterm birth, Inflammation, Innate immune response, Innate lymphoid cells, Preterm labor

## Abstract

**Background:**

Preterm birth (PTB) is one of the major causes of neonatal morbidity and mortality worldwide. It is commonly accepted that the act of giving birth is the final step in a proinflammatory signaling cascade, orchestrated by an intrauterine milieu coupled to hormonal cues. Consequently, the inflammatory process plays a pivotal role during the pathogenesis of human labor, both in term and preterm deliveries. The ability of innate lymphoid cells (ILCs) to act as pro-inflammatory mediators arose the interest to study their role in normal and pathological pregnancies.

The aim of this work was to analyze the relative frequencies of ILCs subsets in pregnancy and the levels of IL-4, IL-17, IL-22, and IFN-γ as inflammatory mediators. Accordingly, we hypothesized that changes in the proportions of ILCs subpopulations could be related to preterm birth.

**Methods:**

We analyzed 15 full-term delivery samples and six preterm delivery samples. In the full-term group (FTB) peripheral blood was taken during routine blood analysis, on 3 occasions: 1st, 2nd and 3rd trimester. After delivery, peripheral blood, cord blood and placenta were collected. In PTB group, peripheral blood samples were obtained on two occasions: before and 24 h after treatment with progesterone. We used flow cytometry to analyze ILCs in maternal peripheral blood, placenta, and cord blood samples. Maternal peripheral blood and cord blood samples were analyzed by enzyme-linked immunosorbent assay for IL-4, IL-17, IL-22, and IFN-γ plasma levels at the time of labor.

**Results:**

We observed significantly increased relative frequencies of ILC2 and ILC3 in the decidua, as well as an increase of ILC2 in cord blood samples in PTB group, compared to FTB samples. We also found a decrease in IFN-γ in peripheral blood samples of the PTB group, suggesting a functional withdrawal. Additionally, IL-4, IL-17, IL-22 levels were similar in PTB and FTB groups, denoting a relevant role in mediating labor.

**Conclusion:**

Our results suggest that ILC2 and ILC3 play a role in PTB by mediating an inflammatory response. Further work is necessary to evaluate the importance of ILCs in the regulation of labor.

## Background

In the past 5 years, an increasing number of studies have focused on a relatively new set of cells, termed innate lymphoid cells (ILCs). Commonly characterized as belonging to the innate component of the immune system, these cells are potentially of substantial interest for neonatal care, mainly for women who suffer a preterm birth (PTB).

PTB is one of the major causes of neonatal morbidity and mortality worldwide. In Portugal, of the 85,500 live-born children in 2015, 8% were born premature [1]. These numbers justify the growing scientific interest in this research, as the world eagerly awaits a new standard of care that might be implemented to allow a reduction in neonatal mortality due to PTB.

The gestational period is comprised of decidualization, placentation and fetal development and requires uterine quiescence and the production of both anti- and proinflammatory cytokines from maternal and fetal cells [2].

Indeed, throughout pregnancy, three distinct biological periods characterized by different immunological microenvironments occur: the first trimester comprises an inflammatory period; while the second trimester comprises an anti-inflammatory period (during which the fetus grows); and the third trimester also comprises an inflammatory period that leads to delivery [3].

Similarly supported by previous work from our group [4, 5], progesterone has been shown to play an important role in reproductive health for the initiation and maintenance of pregnancy, with good results in the prevention of spontaneous abortion and recently in preterm labor [6]. Considering the anti-inflammatory effect of progesterone, we aim to study its effects on preterm birth, through the action on ILCs. Indeed, ILCs appear to be an interesting population to investigate, and reports on ILCs diverse functions that range from inflammation, protection against infections and microorganisms, tumor surveillance and mediation of graft-versus-host disease have been published [7]. As ILCs are thought to play a role in the innate immune response [8], the notion that they may be the basis of a composed inflammatory cascade has drawn our attention. In fact, ILCs can be divided into five distinct groups: NK cells known to produce IFN-γ; Group 1 (ILC1) also known to produce IFN-γ a Th1 like cytokine; Group 2 (ILC2) are characterized by the expression of transcription factor Gata3 and the ability to produce Th2 like cytokines; Group 3 (ILC3), known to produce IL-22 and IL-17; and LTis, important in secondary lymphoid organ formation [8, 9]. ILC3 can be further divided based on the presence of the natural cytotoxic receptor (NCR) NKp44; ILC3 NCR^+^ produce IL-22, while ILC3 NCR^−^ produce IL-17 [7, 10] and both subsets have been found in the decidua [11].

According to the literature, studies performed in humans and mice, suggest ILC3 cells may be of substantial interest in women delivering PTB since they not only express natural cytotoxic receptors (NCR, which is thought to have a stimulatory effect on natural killer (NK) cell action), but also produce IL-17 and IL-22, under the control of the transcription factor RORγt, reviewed by *van de Pavert* et al. [12] and *Spits* et al. [13] respectively and aryl hydrocarbon receptor (AHR) [14]. In this context, IL-17 is substantially relevant since it promotes a proinflammatory state, which might trigger premature birth.

Additionally, IL-22 initiates cell cycle progression via the JAK/STAT pathway, thus contributing to tissue regeneration and homeostasis, reviewed by *Lamarthée* et al. [15], important for placental maintenance.

Studies describing ILCs populations during pregnancy or their role in PTB are still scarce. Vacca et al. [11] published an extensive study that clearly identifies ILC1 and ILC3 populations in first trimester decidua. They demonstrated that these cells have the ability to produce proinflammatory cytokines, including IL-8, IL-22, IL-17A, TNF, and IFN-γ [11], and concluded that ILC3 and ILC1 may play key roles in the implantation process, together with tissue remodeling functions, due to secretion of proinflammatory and angiogenic factors. Conversely, Xu et al. [16] showed that ILC2 is the most abundant population in the decidua, capable of producing Th2-type cytokines, such as IL-4, IL-5, and IL-13. In that study, the authors suggest that the proinflammatory proprieties of ILC2 might underlie the pathological process prompting PTB [16]. Specifically, Xu et al. [16] argue that ILCs populations dynamically change throughout pregnancy. In fact, they also detected ILC3 in the decidua *parietalis*, capable of producing IL-17 and IL-22, suggesting that these cells may be responsible for inflammation-driven PTB. However, Xu et al. [16] also propose that ILC3 can regulate inflammatory actions via the production of IL-13.

The discrepancies in the previously published studies highlight the fact that further studies are needed to clarify the role of ILCs and the innate immune system in normal pregnancy and in abnormal situations, such as PTB. Thus, this pilot study aimed to isolate and characterize ILCs populations from the decidua basalis of pregnant women and compare the results with those in preterm birth samples. Based on the changes in the different ILCs population numbers, we postulate that ILC2 and ILC3 might play a preponderant role in the instigation of labor.

## Results

In the current investigation, we analyzed 15 full-term delivery samples and six spontaneous preterm delivery samples. In the full-term group, the median maternal age was 34 years (33 < 95% CI < 37), the median gestational age at delivery was 40 weeks (39 < 95% CI < 40), the median birth weight was 3635 g (2962 < 95% CI < 3755), and the median placental weight was 500.5 g (448 < 95% CI < 603). In the preterm labor group, the median maternal age was 32 years (22 < 95% CI < 35), the median gestational age at delivery was 36 weeks (34 < 95% CI < 37), the median birth weight was 2505 g (2130 < 95% CI < 3125) and the median placental weight 472 g (413 < 95% CI < 731); these data are summarized in Table 1. All women in this study were nonsmokers. In the clinical information we found a significant statistical difference in birth weight, which is lower in the study group (*p* < 0.001 t-test, 95% CI).

**Table 1:** Descriptive statistics of study and control group. We found a significant statistical difference in birth weight, which is lower in the study group (* *p* < 0.001 t-test, 95% CI)

	Median Maternal Age (Years)	Median Gestational Age (Weeks)	Median Birth Weight (Grams)*	Median Placenta Weight (Grams)
**Full term birth (*n*** **= 15)**	34 (33 < 95% CI < 37)	40 (39 < 95% CI < 40)	3635 (2962 < 95% CI < 3755)	500.5 (448 < 95% CI < 603)
**Preterm birth (*n*** **= 6)**	32 (22 < 95% CI < 35)	36 (34 < 95% CI < 37)	2505 (2130 < 95% CI < 3125)	472 (413 < 95% CI < 731)

**Fig. 1:**
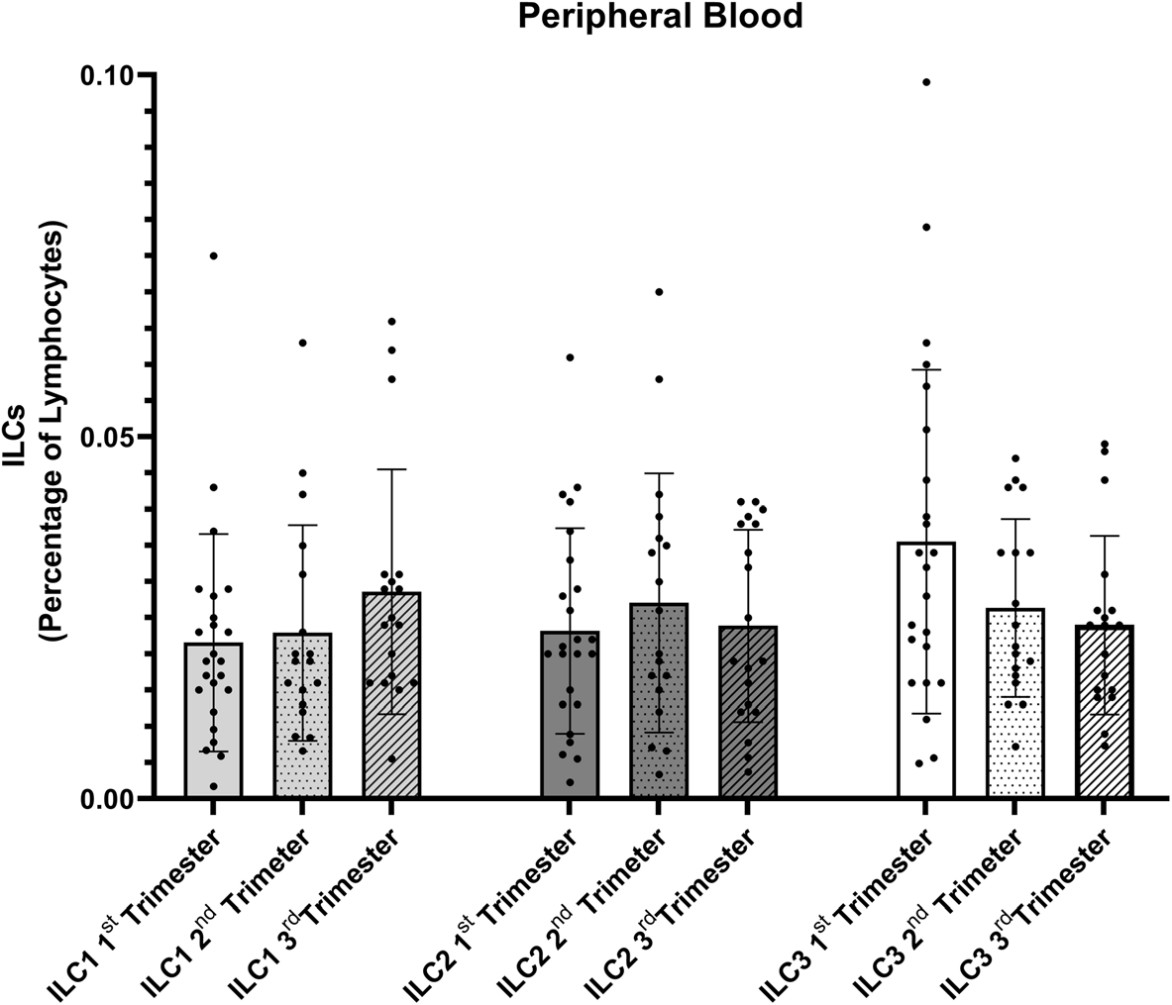
Graphic displaying the relative percentage of the different ILC populations in Peripheral Blood samples in pregnant women in the 1st (*n* = 24), 2nd (*n* = 18) and 3rd (*n* = 18)

**Fig. 2:**
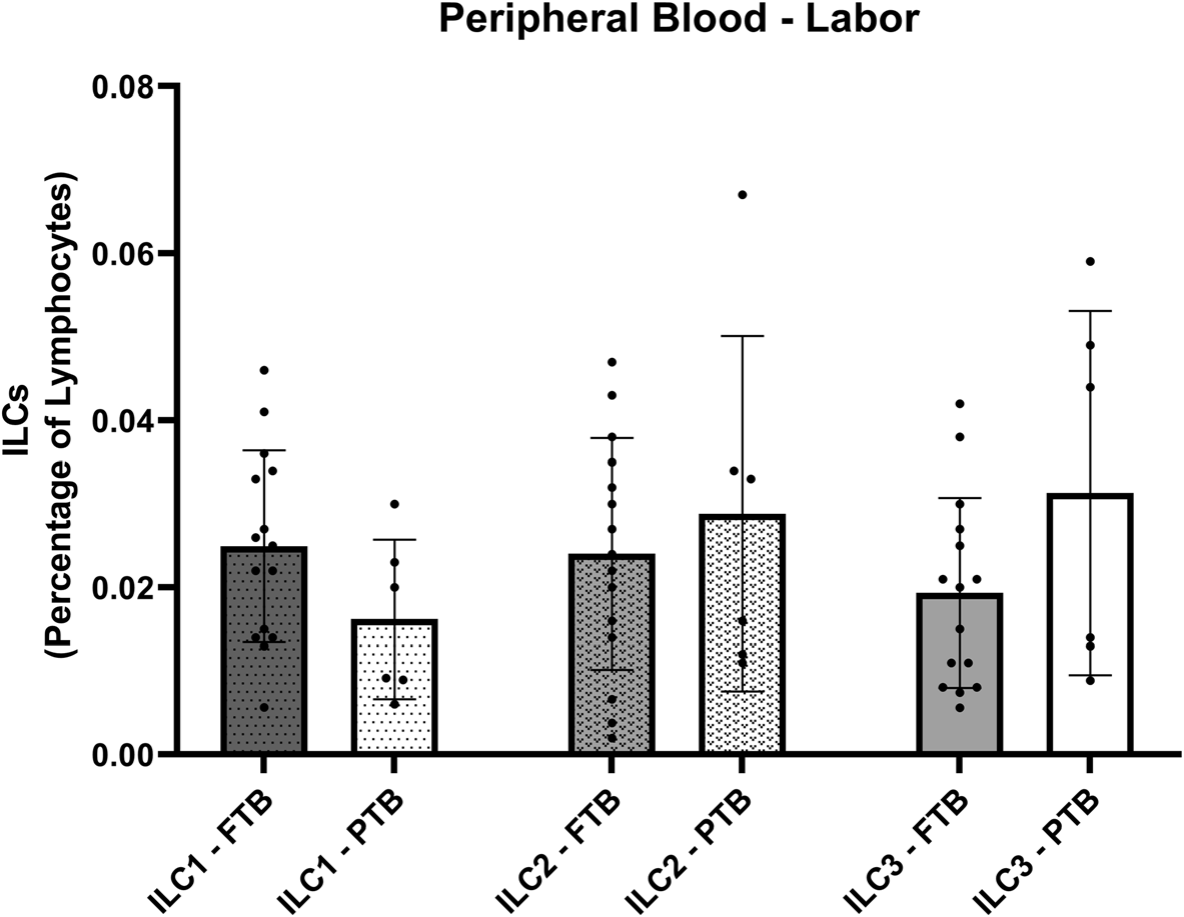
Graphic displaying the relative percentage of the different ILC populations in Peripheral Blood samples in the moment of labor in full term birth (FTB, *n* = 15) compared to preterm birth (PTB, *n* = 6)

**Fig. 3:**
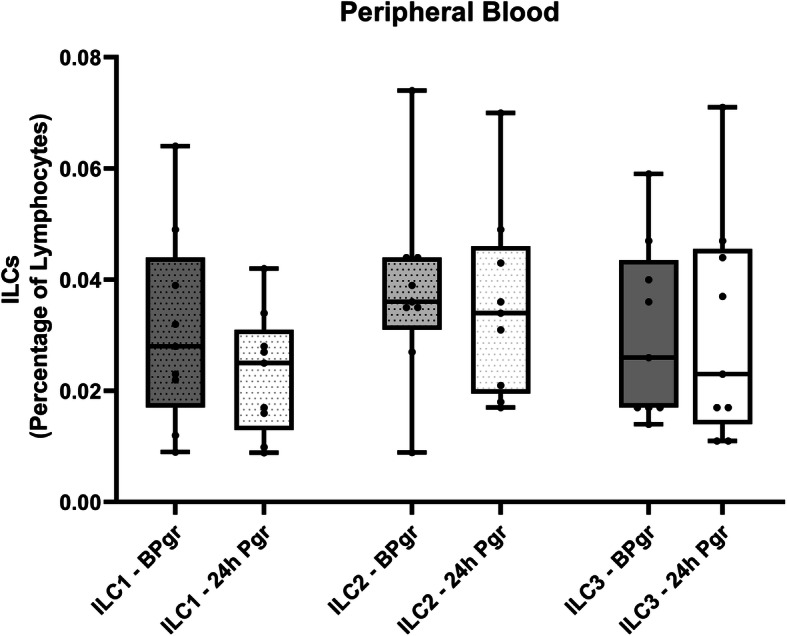
Graphic displaying the relative percentage of the different ILC populations in Peripheral Blood samples before (BPgr) or after the administration of Progesterone (24 h Pgr) in women who delivered PTB

In our investigation, we found no differences in ILCs populations relative frequencies in peripheral blood in FTB group during the first, second or third trimester (Fig. 1), as well as, in the moment of labor (Fig. 2). In addition, we found no differences in ILCs numbers before or after the administration of Progesterone in women who delivered PTB (Fig. 3). This latter result may be explained in part by Progesterone exerting its effects only locally [17], and by the fact that ILCs (namely NK cells) have shown functional and phenotypic differences according to tissue location [18, 19].

**Fig. 4:**
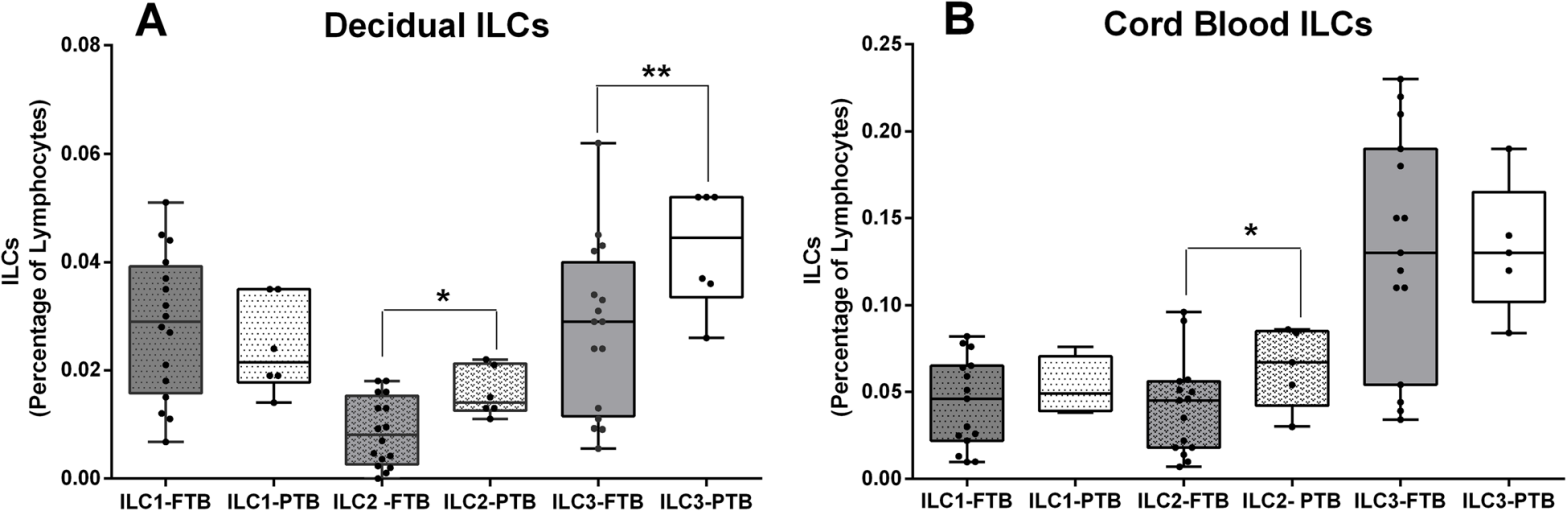
Decidual and cord blood ILCs in FTB and PTB. **a** Graphic displaying the relative percentage of the different ILC populations in full term birth (FTB, *n* = 15) compared to preterm birth (PTB, *n* = 6), in human decidua. **b** Graphic displaying the relative percentage of the different ILC populations in FTB (*n* = 15) compared to PTB (*n* = 5) in cord blood samples. Multiple t-student tests where used for statistical analysis with a 95% confidence interval, *p*-value * *p* < 0.05; ** *p* < 0.01 (two tailed)

In our study, there was a significant increase in ILC2 and ILC3 frequencies in the decidua of women who delivered preterm (*p* < 0, 05 t-test, 95% CI) (Fig. 4a); in cord blood samples we verified an increase in ILC2 frequency also in that group (*p* < 0, 05 t-test, 95% CI) (Fig. 4b). When observing the ILC3 subsets, ILC3 NCR- and ILC3 NCR+ cells within the FTB and PTB group, the ILC3 NCR- population was clearly increased, representing the predominant ILC3 subset (*p* < 0.001, student t-test 95% CI) (data not shown).

**Fig. 5:**
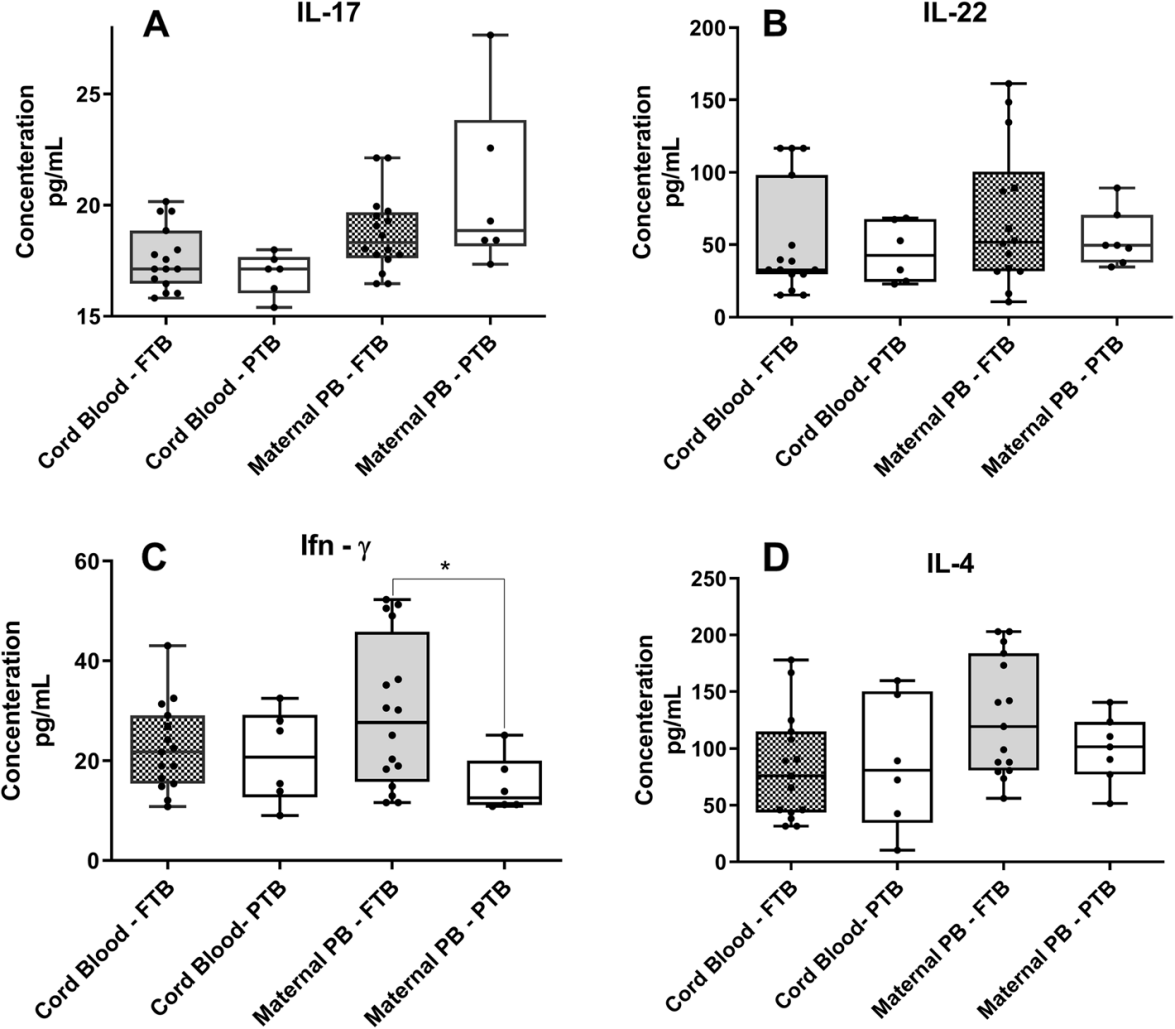
Enzyme-Linked Immunosorbent Assay (ELISA) in cord blood and maternal peripheral blood in FTB and PTB. **a** Graphic displaying IL-17 plasmatic concentrations in cord blood FTB (*n* = 15) and PTB (*n* = 5), as well as, plasmatic concentrations of IL-17 in maternal Peripheral Blood (PB) (*n* = 6). **b** Graphic displaying IL-22 plasmatic concentrations in cord blood FTB (*n* = 15) and PTB (*n* = 6), as well as, plasmatic concentrations of IL-22 in maternal Peripheral Blood (PB) (*n* = 6). **c** Graphic displaying Ifn-γ plasmatic concentrations in cord blood FTB (*n* = 15) and PTB (*n* = 6), as well as, plasmatic concentrations of Ifn-γ in maternal Peripheral Blood (PB). **d** Graphic displaying IL-4 plasmatic concentrations in cord blood FTB (*n* = 15) and PTB (*n* = 6), as well as, plasmatic concentrations of IL-4 in maternal Peripheral Blood (PB). Student’s t-tests were used for statistical analysis with a 95% confidence interval. A statistical significant decrease in Ifn-γ plasma concentration was found, in peripheral blood samples in women with PTB

Determination of, IL-17, IL-22 and IL-4 plasma levels in cord blood and peripheral blood samples showed no differences between FTB and PTB groups (Fig. 5a, b and d respectively). Moreover, we found reduced levels of IFN-γ in peripheral blood samples of women who delivered PTB (Fig. 5c).

## Discussion

These preliminary results demonstrate that ILCs are present in the decidua of both FTB and PTB pregnant women, since we clearly detected them at the maternal-fetal interface, as well as in cord blood samples.

ILCs, specifically the ILC2 and ILC3 populations, have been described to play a preponderant role in pregnancy [16, 20]. Moreover, Xu et al. [16] reported that ILC3 cells can also produce IL-13 (a proinflammatory cytokine), which, if confirmed, could also contribute for the instigation of labor. Additionally, as mentioned by *Mjösberg and Spits* [7]*,* ILC3 NCR^+^ cells produce mainly IL-22, while NCR^−^ cells produce IL-17 [7], which is intriguing since IL-22 is considered a homeostatic cytokine that contributes to tissue and organ integrity. On the other hand, IL-17 mostly functions as a proinflammatory cytokine [21]. Herein, we found the ILC3 NCR^−^ to be the dominant ILC3 population, which is in accordance with the findings highlighted by Vacca et al. [11]. Undeniably, ILC3 population can produce proinflammatory cytokines in deciduas isolated from women in their first trimester of pregnancy, highlighting a potentially important role for ILC3 cells in the first stages of pregnancy. These facts support our results revealing elevated ILC2 and ILC3 frequencies in decidua of women that underwent PTB, as well as IL-17 concentration found in our ELISA experiments. Indeed, the concentrations determined on labor day in the FTB group were like those found in the PTB group, denoting an inflammatory role of IL-17 in the instigation of labor. Regarding IL-22 levels, a work carried out by Perfetto et al. [22] suggests that IL-22 may play a key role in maintaining decidual homeostasis and help to constrain inflammation by contracting the effect of IL-17. These authors found lower levels of IL-22 in unexplained recurrent pregnancy loss in human decidua. Again, the fact that we found comparable values of IL-22 plasma concentrations in PTB and FTB groups, may reflect a loss of the protective role of IL-22 and the prevalence of the pro-inflammatory effect of IL-17.

The discovery of reduced plasma levels of IFN-γ in peripheral blood samples of women who delivered PTB is noteworthy. A study by Hanna N et al. [23] demonstrated that IFN-γ represses COX-2 expression and PGE2 production in human placental samples from both term and preterm labor deliveries, suggesting that functional withdrawal of IFN-γ may be involved in the onset of term or preterm labor. Since our results are from peripheral blood samples, further work needs to focus on decidual samples to identify IFN-γ producing ILCs, namely ILC1 or NK cells.

Nonetheless, we cannot reject the assumption that an exacerbated Th2-type response in PTB may be orchestrated by ILC2. The comparable values of IL-4 plasma levels found in the FTB and PTB groups support this hypothesis. Moreover, ILC2 are capable of presenting antigens to T CD4^+^ cells and induce proliferation towards a Th2 phenotype in an IL-2 dependent manner [24]. This is a remarkable finding since it proposes a crosstalk between the innate and adaptive immune systems [25–27]. However, ILC3 have been shown to express MHC class II molecules, promoting a T cell-mediated response [28]. This fact, may elucidate the role of ILCs in mounting an adaptive immune response, potentially representing a tolerance mechanism towards the fetus, which is of substantial interest. A strong point of this study is its corroboration with existing hypothesis that an inadequate inflammation triggered immunological response prompts PTB, suggesting a key role of ILCs in this process; unfortunately, numerous mechanisms underlying ILCs actions in pregnancy remain to be ascertained. The data herein presented suggests that in PTB group there is an inflammatory response orchestrated by an elevation of ILC2 and ILC3. However, this group is being treated with progesterone, which is manly an anti-inflammatory hormone. To explain this discrepancy, further work is needed to ascertain the expression of progesterone receptors in the different ILCs populations. In fact, previous work conducted by *Areia* et al. [5] demonstrated a decline in T regulatory cells (Treg), positive for membrane progesterone receptor (mPR^α+^), theorizing a reduction in progesterone anti-inflammatory action through Treg mPR^α+^ cells. Our study does present some limitations that must be considered. Because our small sample size, the results should be interpreted with caution. To address this issue, future studies should include a larger number of women, with particular attention to a comprehensive analysis of cytokine release throughout pregnancy.

## Conclusion

The data presented herein suggest that labor might be characterized by decreased tissue remodeling and repair functions, accompanied by a marked inflammatory response, due to high levels of ILC2 and ILC3. Also, our ELISA experiments propose that labor might be characterized by a functional INF- γ withdrawal. However, there was no significant decline in ILC1 population that could account for this result.

Nevertheless, our results are encouraging, as they suggest a role of ILCs in the regulation of labor.

Thus, we aimed to expand the current knowledge on the immunology of pregnancy by focusing on ILCs fluctuations during pregnancy and meticulous analysis of cytokine profile.

## Methods

The present study was approved by Coimbra Hospital and Universitary Centre ethics committee. Signed informed consents were obtained from all patients whom their blood samples, placentas and clinical data were used in this study. All methods involving human participants, human sera and human data were carried out in accordance with Declaration of Helsinki and approved by the Faculty of Medicine from Coimbra Univaersity.

### Population

Female patients who planned to deliver at the Obstetric Department of Coimbra Hospital and Universitary Centre (CHUC) were invited to participate in the study.

The study inclusion criteria consisted of pregnant women monitored by normal prenatal appointments and women presenting to the emergency room in labor. Inclusion criteria for full term birth group (FTB) comprised: healthy pregnant women attending normal prenatal appointments; full term singleton pregnancies, delivered after spontaneous labor; and first prenatal appointment before 14th week gestation. The inclusion criteria for the preterm birth (PTB) group were as follows: admission to the Fetal Maternal Medicine Obstetric Department of CHUC with confirmed spontaneous preterm labor, singleton pregnancy, gestational age between 24 weeks + 0 days and 36 weeks + 6 days, intact membranes, cervical length ≤ 25 mm and the use of Atosiban (competitive oxytocin receptor antagonist) for tocolysis (for contraction cessation). Administration of natural progesterone was done after tocolysis with Atosiban, vaginally, once daily, in a 200 mg dosage.

Exclusion criteria were the following: multiple gestation; preterm rupture of membranes; chorioamnionitis; placenta previa or placental abruption; intrauterine growth restriction; and pre-existent maternal diseases, namely: hypertension, diabetes, autoimmune diseases, and allergies. Clinical chorioamnionitis was diagnosed based on histologic evaluation and clinical laboratorial parameters like fever, maternal tachycardia, fetal tachycardia, maternal leukocytosis, uterine tenderness, foul-smelling amniotic fluid; elevated maternal C-reactive protein and/or amniotic IL-6.

Women subjected to elective pre-labor Caesarean section were not included as they have other medical etiologies, not focused on this work.

### Isolation of innate lymphoid cells

In the FTB) peripheral blood was taken, during routine blood analysis on 3 occasions: 1st, 2nd and 3rd trimester. Cells were isolated using a Ficoll-paque™ gradient and stained as further discussed for flow cytometry analysis. After delivery, peripheral blood, cord blood and placenta were collected. In PTB, peripheral blood samples were obtained on two occasions: before and 24 h after treatment with progesterone. In this sense, a correlation was determined in the ILCs population studied, because of progesterone administration. After delivery, peripheral blood, cord blood and placenta were collected.

**Fig. 6:**
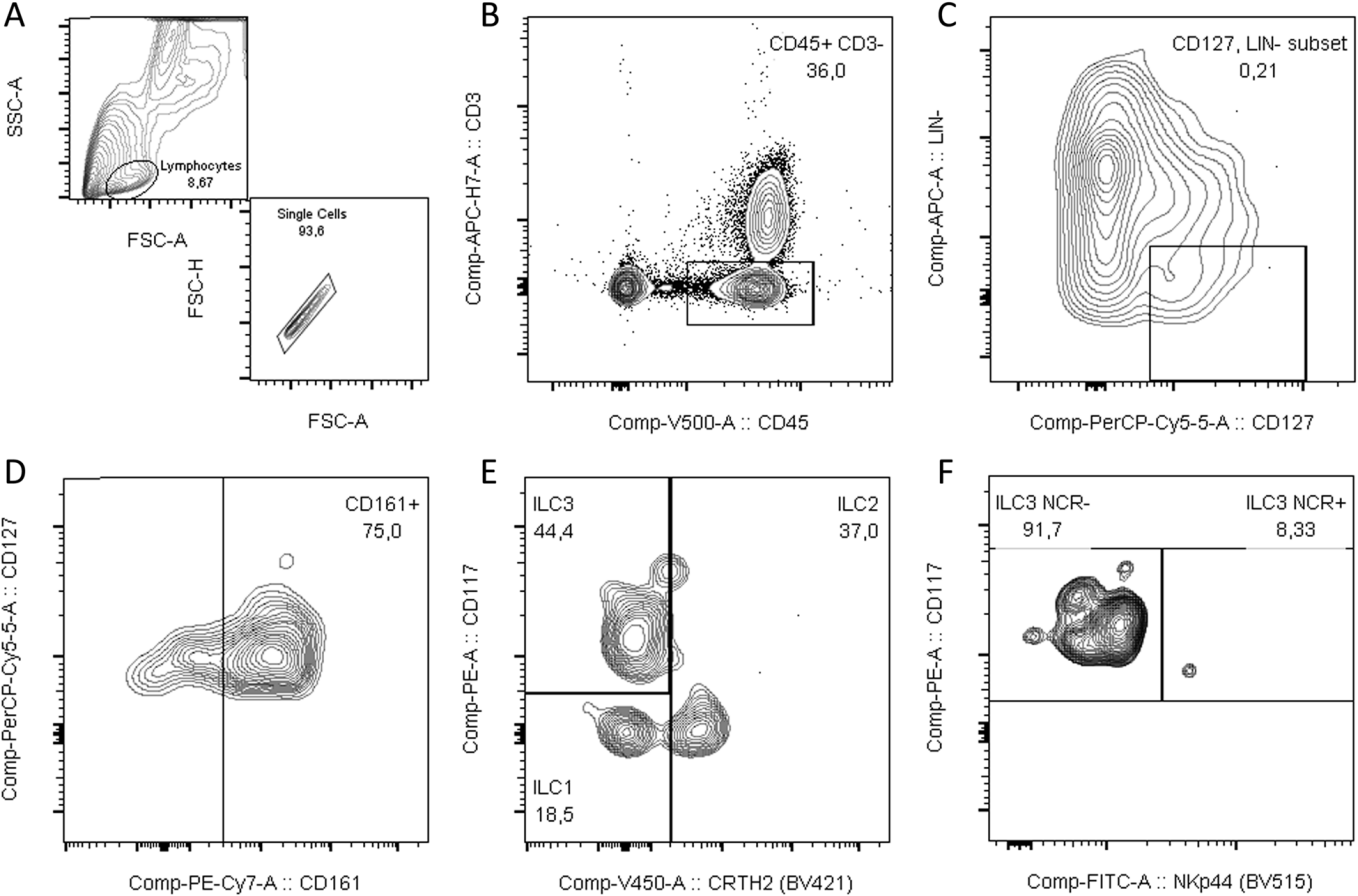
Gating strategy for identification of ILC3 subpopulations. **a** Identification of lymphocyte population. **b** Gating of CD45 + CD3-cells. **c** Selection Lin-CD127+ cells (**d**). isolating CD161+ cells. **e** Gating ILC3 cells as CRTH2- CD117+. **f** Discrimination between ILC3 NCR+ and ILC3 NCR- based on NKp44 expression (Data analyzed in FlowJo®)

**Fig. 7:**
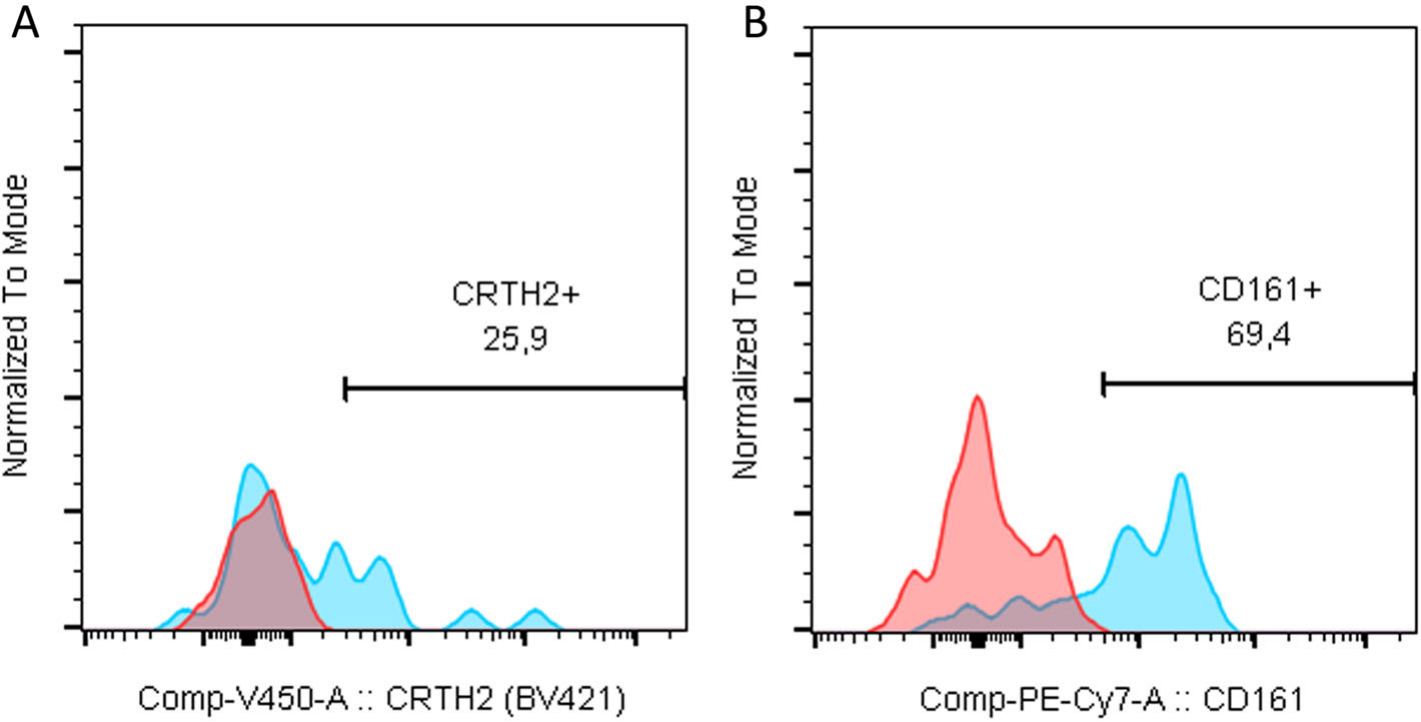
Full minus one (FMO) for CRTH2 (**a**) and FMO for CD161 (**b**)

In both PTB and FTB groups, the placenta was rinsed in phosphate buffered saline (PBS, Ca^2+^- and Mg^2+^-free) (Corning®, New York, USA), to wash cloths and superfluous blood. Decidual tissue was dissected while soaking in 1× PBS (Ca^2+^- and Mg^2+^-free). Lymphocytes were isolated from the decidua basalis and adjacent tissue (villi) as described by Yi Xu et al. [29]. Cells were counted on a Beckman Coulter AcT Diff automatic cell counter (Beckman Coulter, Brea, California, EUA), and a 100 μL cell suspension containing 1 × 10^6^ isolated lymphocytes was placed in a cytometry tube and labelled with primary antibodies (BD Biosciences, San Jose, USA). For lymphocyte discrimination, CD45^+^ and CD3^−^ were used. Lineage-negative (Lin^−^) cells were labeled with CD1 (clone HI149), CD11c (clone B-ly6), CD34 (clone 581), CD123 (clone 7G3), TCRγδ (clone xB1), TCRαδ (clone T10B9), BDCA2 (clone 201A), FcER1 (clone AER-37), CD19 (clone HI149), CD14 (clone M5E2), and CD94 (clone HP-3D9) and discriminated against CD127 (clone A019D5). Cells expressing CD161 (clone HP-3G10) were then selected. To ascertain the different ILC populations, CD117 (clone 104D2), CRTH2 (clone BM16) and NKp44 (clone p44–8) antibodies were used as described by Hazenberg et al. [30]. Gating strategy can be viewed in Figs. 6 and 7. Stained samples were acquired on a BD FACS Canto II flow cytometer (BD Biosciences, San Jose, CA, USA) equipped with 3 lasers to allow multicolor detection with different fluorophores, using BD FACSDiva v.6.1.3 software (BD Biosciences, San Jose, USA). All samples were then analyzed with FlowJo v.10.7 software (Tree Star Inc., Ashland, OR, USA).

### Enzyme-linked Immunosorbent assay (ELISA)

For IL-17, IL-22, IFN-γ and IL-4 ELISA determination we used Biolegend Legend Max™ ELISA Kits. Peripheral whole blood and cord blood was collected in the moment of labor, to a 6 mL EDTA tube. To separate plasma from whole blood, tubes were centrifuged for 15 min at 1000 g. Samples were stored in 200 μL aliquots at − 80 °C to prevent repetitive freeze/thaw cycles. One hundred microliter of plasma was used in triplicate and absorbance was determined using a Bio-Rad® model 600 microplate reader, according to manufacturer protocols (Bio-Rad, Hercules, CA, USA). Average of triplicate readings was performed, and a standard curve was generated using a four-parameter logistic curve-fit to determine plasma concentrations in pg/mL.

### Statistical analysis

Each data set was analyzed using student t-test analysis with a confidence interval of 95%. Statistical analysis was performed using GraphPad Prism, version 7 (GraphPad Software, Inc., La Jolla, CA, USA). Differences were considered statistically significant at a *P* value of < 0.05 and are annotated as follows: * *p* < 0.05; ** *p* < 0.01; *** *p* < 0.001 and **** *p* < 0.0001.

## Data Availability

The datasets analyzed during the current study are available from the corresponding author on reasonable request.

## Abbreviations

FTB: Full-term birth
PTB: Preterm birth
IFN-γ: Interferon gamma
IL: Interleukin
ILCs: Innate lymphoid cells
NCR-: Natural cytotoxic receptor
PGE2: Prostaglandin E2
RORγt: Retinoic acid receptor-related Orphan nuclear Receptor gamma
